# Supplementary material for: Multi-Omics Analysis Reveals Up-Regulation of APR Signaling, LXR/RXR and FXR/RXR Activation Pathways in Holstein Dairy Cows Exposed to High-Altitude Hypoxia
Source: Animals (Basel). 2019 Jul 1;9(7):406. doi: 10.3390/ani9070406 (PMC6680605; doi:10.3390/ani9070406)
Supplement: Supplementary file 1 [file animals-09-00406-s001.pdf]

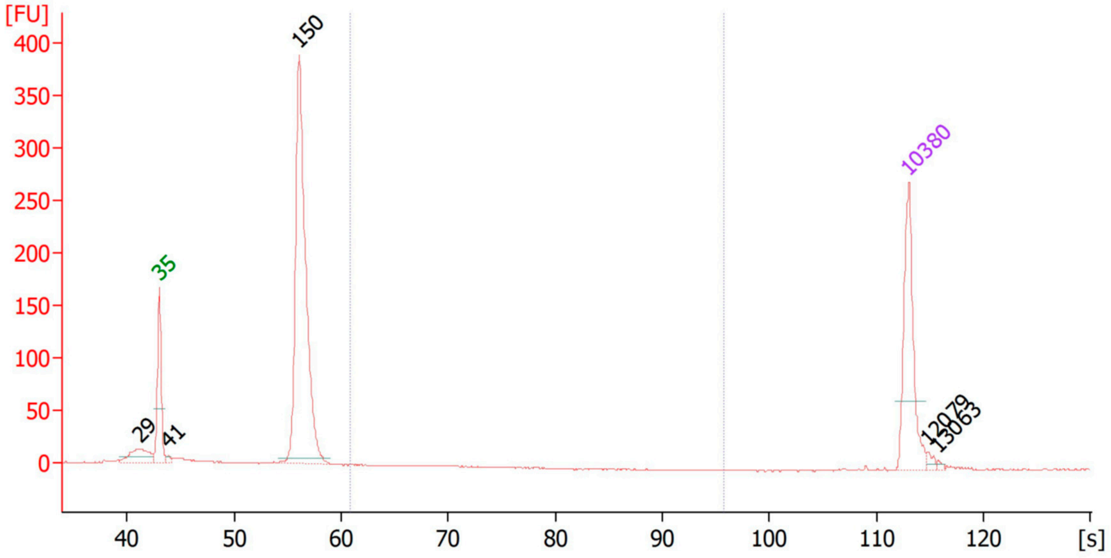

Overall Results for sample: 160923A_ (HA group)

Number of peaks found: 5

Noise: 0.4 Corr. Area 1: 0.0

Peak table for sample : 160923A_(HA group)

Peak	Size [bp]	Conc. [pg/µl]	Molarity [pmol/l]
1	29	0.00	0.0
3	41	5.03	188.0
4	150	460.38	4653.7
6	12079	0.00	0.0
7	13063	0.00	0.0

Region table for sample 160923A_ (HA group)

From [s]	To [s]	Corr. Area	% of Total	Average Size [bp]	Size distribution in CV [%]	Conc. [pg/µl]	Molarity [pmol/l]
60.82	95.77	0.0	0	211	0.1	0.02	0.2

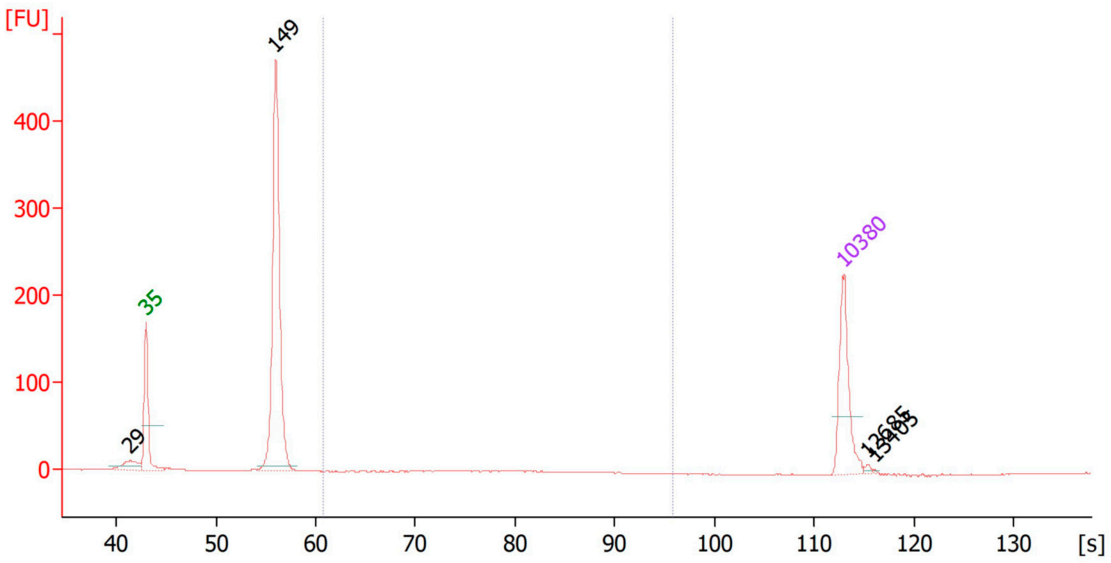

Overall Results for sample: 160923A_ (SL group)

Number of peaks found: 4

Noise: 0.4 Corr. Area 1: 0.1

Peak table for sample : 160923A_(SL group)

Peak	Size [bp]	Conc. [pg/µl]	Molarity [pmol/l]
1	29	0.00	0.0
3	149	464.08	4710.1
5	12685	0.00	0.0
6	13405	0.00	0.0

Region table for sample 160923A_ (SL group)

From [s]	To [s]	Corr. Area	% of Total	Average Size [bp]	Size distribution in CV [%]	Conc. [pg/µl]	Molarity [pmol/l]
60.82	95.77	0.0	0	402	14.5	0.06	0.2
